# Nursing staff fluctuation and pathogenic burden in the NICU - effective outbreak management and the underestimated relevance of non-resistant strains

**DOI:** 10.1038/srep45014

**Published:** 2017-03-21

**Authors:** Kai O. Hensel, Rhea van den Bruck, Ingo Klare, Michael Heldmann, Beniam Ghebremedhin, Andreas C. Jenke

**Affiliations:** 1 HELIOS University Medical Center Wuppertal, Department of Pediatrics and Neonatology, Center for Clinical and Translational Research (CCTR), Witten/Herdecke University, Germany; 2 Department of Neonatology, University Hospital Essen, Germany; 3 Robert-Koch Institute, Wernigerode, Germany; 4 HELIOS University Medical Center Wuppertal, Institute of Medical Laboratory Diagnostics, Center for Clinical and Translational Research (CCTR), Witten/Herdecke University, Germany; 5 EKO Children' Hospital, Oberhausen, Witten/Herdecke University, Germany

## Abstract

In the course of a hospital management takeover, a microbial outbreak took place in a tertiary neonatal intensive care unit (NICU). Here, we characterize the outbreak and its management. About 4 months prior to takeover, there was a sharp increase in positive isolates for MSSA and multidrug-resistant organisms (MDROs). Simultaneously, the nursing staff sick leave rate increased dramatically which directly correlated with the number of infection/colonization per week (r^2^ = 0.95, p = 0.02). During the following months we observed several peaks in positive isolates of methicillin-sensitive staphylococcus aureus (MSSA), MDROs and subsequently a vancomycin-resistant enterococcus (VRE) outbreak. Interventional outbreak management measures were only successful after substantial recruitment of additional nursing staff. None of the VRE, but 44% (n = 4) of MDRO and 32% (n = 23) of MSSA colonized infants developed symptomatic infections (p = 0.02). Among the latter, 35% suffered from serious consequences such as osteomyelitis. The most important risk factors for colonization-to-infection progression were low gestational age and birth weight. Nursing staff fluctuation poses a substantial risk for both bacterial colonization and infection in neonates. Comprehensive outbreak management measures are only successful if adequate nursing staff is available. Non resistant strains account for most neonatal infections – possibly due to their limited perception as being harmful.

In recent times both pediatric and adult hospitals have been facing the challenge to handle ever-increasing patient throughputs, mainly for economic reasons^1^. In contrast, medical innovations such as personalized medicine, dedicated communication and maintenance of highest hygienic standards are extensively time- and cost-consuming. Naturally, this discrepancy oftentimes results in a decreased supply of a *prima facie* less measurable medical service, namely nursing staff employment. Even though this issue has been labeled as a “massive global health care problem” by the WHO over a decade ago, to date it still has not been adequately addressed^2^,^3^. The problem has gained importance to a dimension that has led to the introduction of the term “healthcare-associated infection” which is now regularly used^4^.

As a result, the nosocomial spread of multidrug-resistant bacteria on intensive care units has increased to an alarming extent^5^. In adult medicine the composition of the nursing staff has been shown to be related to the incidence of primary blood stream infections in hospitalized patients^6^. Furthermore, the association of limited nurse staffing in the intensive care unit and poor clinical patient outcomes are consistent with findings in studies of the general acute care adult population. However, little is known about the effects of nursing staff composition in neonatal medicine.

In addition to the general problem of healthcare-associated infections, the incidence of multidrug-resistant organisms (MDROs) has increased over the last years in the neonatal intensive care units^7^. Currently, the two most commonly encountered organisms are vancomycin-resistant enterococci (VRE) and methicillin resistant *S. aureus* (MRSA). Major risk factors for neonatal VRE colonization could have been identified as low birth weight, prematurity, and long-term antimicrobial therapy^8^. On the other side, the backgrounds of MRSA colonization in the neonatal intensive care unit (NICU) are still largely unknown^9^. Strikingly, even though the ongoing scientific discussion is mainly focused on MDROs, infections with non-resistant bacteria - particularly *Staphylococcus aureus* – are a major problem on NICUs. In fact, *S. aureus* is still the second most common cause of central line-associated bloodstream infections; especially in very low birth weight (VLBW) infants^10^ due to their immature immune system and thus attenuated neutrophil response and activity^11^,^12^.

Between January and April 2015 the managing company of the NICU at the St. Anna Hospital Wuppertal, Germany, changed. Prior to this takeover, no outbreak of any kind (minor of major) of VRE, MRSA or methicillin sensitive *S. aureus* (MSSA) colonization or infection was recorded over a period of greater than 10 years. In the course of the recent hospital takeover however, significant nurse understaffing and a microbial outbreak took place at the NICU. The aim of this study was to characterize the outbreak with regard to molecular and epidemiological characteristics, to identify potentially causal risk factors, to evaluate the effectiveness of the applied countermeasures and to analyze the pathogenicity of the most commonly featured bacteria.

## Methods

### Hospital characteristics

The outbreak occurred in a 28-bed neonatal unit, including a 10-bed NICU, in a tertiary care hospital in Germany. The NICU comprised two wards at two different locations; one consisting of 4 intensive care and 6 intermediate care beds (primary admission ward at the maternity and obstetrics hospital), the other one included 6 intensive care and 12 intermediate care beds including one isolation room. The neonatal service features approximately 250 annual admissions of neonates born in-house and approximately 50 out-born neonates.

### Epidemiological data

Medical, nursing, and laboratory records of all patients admitted to the neonatal service during the outbreak were reviewed. Anonymized data including date of birth, sex, gestational age, birth weight, maternal underlying disease, applied medications and administration of intravenous/central catheters or nasogastric tubes were collected and analyzed.

### Microbiological analysis

Targeted active surveillance was implemented for all infants hospitalized in the NICU of St. Anna Hospital. Routine screening was intensified from once per week to twice per week and furthermore extended to all infants without well-known risk factors (such as premature rupture of membranes, chorioamnionitis, maternal immune suppressive therapy), e.g. normal deliveries. Rectal swab specimens were streaked on selective chromogenic media which were incubated at 37 °C for 24–48 hours. The colonies were identified as *Enterococcus faecium* by MALDI-TOF mass spectrometry. VRE presence was confirmed by vancomycin Etest (OXOID, Wesel, Germany) according to the EUCAST (www.eucast.org) guidelines. Infection was defined as combination of clinical symptoms and isolation of the respective organism – for example radiological signs of a pulmonary abscess and isolation of MSSA from bronchopulmonary lavage.

### Macrorestriction analysis in pulsed field gel electrophoresis

Genomic DNA was isolated, digested with restriction endonuclease *Sma*I, and treated as previously described^13^. The agarose gel concentration was 1% and the CHEF-DR III apparatus (BIO-RAD laboratories, Hercules, CA, USA) was used for pulsed field gel electrophoresis (PFGE). Selected ramped pulsed times were as follows: 1–11 sec for 15 h and 11–30 sec for 14 h at 14 °C. Relatedness between banding patterns was calculated using a band based similarity coefficient (Dice) and UPMGA clustering (BioNumerics, Applied Maths, Sint-Martens-Latem, Belgium). A composite tree of all 58 hospital VRE of groups I–III resolved in 18 independent PFGE gels was generated the same way with the exception of increasing the value of the “position tolerance setting” to 1.5% (default: position tolerance setting 1.0%; optimization 0.5%). PFGE types were assigned based on >90% similar patterns and additionally considering recommendations for fragment pattern analyses as previously described^14^,^15^.

### Outbreak management

**Figure 1:**
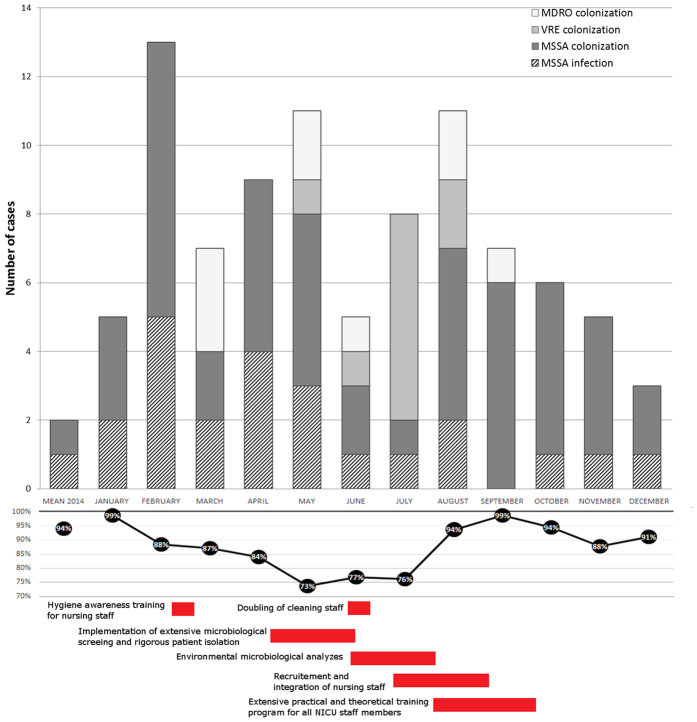
2015 Outbreak and hygienic outbreak control intervention characteristics. Top: Pathogen colonization and infection rate in patients admitted to the NICU; Middle: Available nursing staff, 100% = estimated minimal requirement for adequate patient care according to GNPI guidelines; Bottom: Time-course of applied interventional outbreak management measures.

The outbreak management was coordinated by pediatric infectious disease specialists and hygienists and involved all NICU nurses as well as all physicians of the neonatal department. The essential goal of reestablishing a favorable patient-to-nurse ratio according to the guidelines of the German society for neonatology and pediatric intensive care (GNPI) was achieved by reemployment of qualified NICU nurses^16^. Mandatory training sessions for all involved nurses, neonatologists as well as for the cleaning staff were held by a specialized infection control hygienist and a microbiologist. This included – but was not limited to – repetitive compliance observations regarding hand disinfection and aseptic interventions and, where applicable, corrective trainings. To improve the overall outbreak management compliance, comprehensive procedural instructions with a special focus on hygienic measures were addressed to all involved neonatologists, nurses, cleaning staff, ward assistants and parents and displayed in every patient room of the NICU as well as in public hospital areas. Serial inspections as well as environmental microbiological analyses by the infection control committee took place in the delivery room, the operating theatre and in the NICU. Enhanced outbreak management measures were implemented, including contact precautions for all patients colonized with MSSA, MDROs and VRE as well as standard precautions for non-colonized patients. Cohorting of affected neonates took place by relocating patients colonized with the same multiresistant strain together in a single room. The appropriateness of antimicrobial therapy, especially of glycopeptides and linezolid as part of an antimicrobial regimen (i.e. second-line antimicrobial regimen), was evaluated with NICU physicians and pediatric infectious disease specialists based on the bacterial resistance patterns as evaluated by microbiological analyses. A detailed time-course of the applied sequential outbreak management interventions is outlined at the bottom of Fig. 1.

### Statistical analysis

Data were compared using the Mann-Whitney U test according to normality assumptions on univariate analysis followed by Bonferroni correction for multivariate analyses. Categorical variables were compared using the Fisher exact test. Statistical analyses were performed with GraphPad Prism, version 5.0.

### Ethics statement

The study was carried out in accordance with the declaration of Helsinki and approved by the Witten/Herdecke Ethics Committee. Written informed consents were obtained from legal guardians where appropriate.

## Results

### Outbreak epidemiology and management

**Figure 2:**
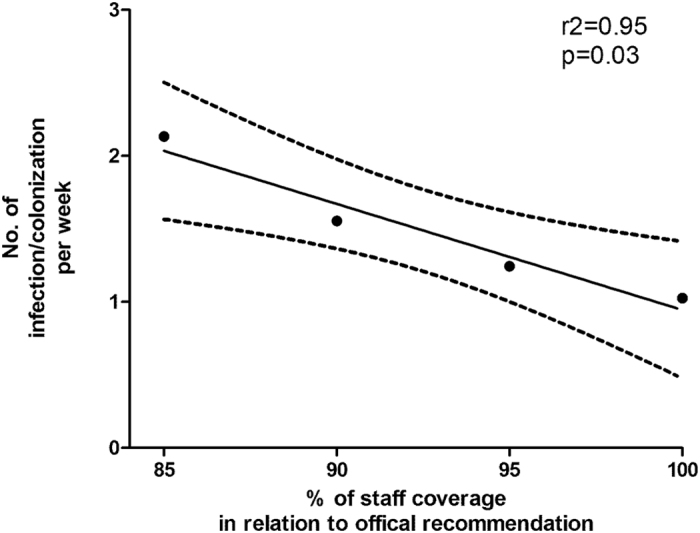
Correlation analysis between nursing staff coverage and incidence of pathogenic infections/colonizations per week. Time periods were categorized depending on nursing staff coverage and correlated with the number of infections and colonizations per week by linear regression analysis.

Between January and December 2015, altogether 266 neonates – among that 54 very low birth weight (VLBW) infants – were admitted to the St. Anna Hospital NICU. Median gestational age was 27.8 weeks (95% confidence interval (CI) 27–28.6 weeks) and median birth weight was 1019 g (95% CI 927–1111). The hospital management acquisition of the St. Anna NICU by a private hospital management company was officially announced in January 2015 and took place April 1^st^ 2015. At this time a substantial increase in colonization rates of several pathogens - multi-resistant strains and MSSA – was observed in neonates admitted to the NICU. Overall, 71 neonates were colonized with MSSA, 10 with VRE and 9 with other MDROs (Table 1). 75.9% of all admitted VLBW infants were colonized with one or more of the following pathogens: VRE (n = 5), MDROs (n = 9) or MSSA (n = 34). Within this time, three major peaks were noted – February, May and August of 2015 (Fig. 1) – which correlated with the number of resignations and percentage of nursing staff absent from work due to sick leave notes or resignation (r^2^ = 0.95, p = 0.03, Fig. 2). Neither did gestational age differ between colonized (median 27.8; 95% CI 26.9–28.7) and non-colonized infants (median 27.4; 95% CI 25.9–28.8), nor did birth weight (median 1039 g, 95% CI 935–1143 vs. 900 g, 95% CI 719–1082). Compared to the pre-takeover period, no change in the patients’ medical management was noted, particularly not with regard to use of antimicrobial agents.

Several steps were implemented promptly during this time in order to control this outbreak. Specifically, this included enhanced infection control measures and audits on antimicrobial and disinfectant use (*methods section,* Fig. 1). Each of these early interventions resulted in a moderate reduction of colonization and infection rates followed by a second subsequent rise in both colonizations and infections (Fig. 1). A substantial recruitment of qualified nursing staff was achieved in late July 2015. This was followed by an extensive hygiene awareness training program from August through October. Ultimately, these measures lead to sustained reduction of colonization and infection rates to a pre-outbreak level in December 2015 (Fig. 1).

### Microbiological analysis revealed all VRE isolates to be the same strain

**Figure 3:**
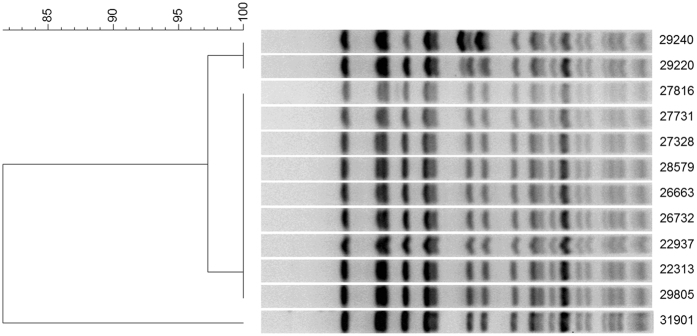
Cluster analysis of VRE isolates based on SmaI-macrorestriction patterns resolved in PFGE. 12 vanA-positive *E. faecium* isolates of NICU patients hospitalized at the neonatology department of the HELIOS University Clinic Wuppertal were positive for the virulence genes *esp* and *hyl*. The dendrogram was evaluated using Dice coefficient and UPMGA clustering (BioNumercis software). The VRE isolates showed identical or highly related banding patterns (>95% identity agreement).

The clonal relationship of the 12 VRE isolates from the NICU outbreak in the summer of 2015 was assigned based on a 90% similarity cut-off and the recommendations as described in the materials and methods section. The genotypic characterization of the glycopeptide resistance for all VRE isolates detected the same alteration in the *vanA* element (Fig. 3). However, the VRE isolates were all sensitive to daptomycin, linezolid, and tigecycline.

### Systemic and localized infections are common events in MSSA and MDRO´s but not in VRE colonization

Lower birth weight and younger maternal age proved to be independent risk factors for colonization with MDROs compared to infants with VRE or MSSA colonization. In addition, only male infants were colonized with MDROs while female neonates remained entirely unaffected. No differences in epidemiological parameters were noted in infants colonized with VRE or MSSA (Table 1). Importantly, none of the VRE colonized patients eventually developed an infection. In contrast, MSSA infections occurred in 32% of MSSA colonized neonates and MDRO infection developed in 44% of MDRO colonized patients (Table 1). Infection with MSSA occurred more often in infants with lower gestational age and lower birth weight. Interestingly, male gender and prenatal antibiotic therapy seemed to be associated with a reduced risk for progression from colonization to infection (Table 2).

Eight of the MSSA infected patients (35%) were affected by systemic infections such as pulmonary abscess, osteomyelitis or septicemia. The other 15 patients (65%) had localized disease such as conjunctivitis or skin abscesses. All patients fully recovered following the application of systemic antibiotic therapy. Infants with lower gestational age and lower birth weight were significantly more likely to have systemic infections. Furthermore, prenatal maternal steroid application was associated with an increased risk for neonatal systemic infection, while on the other hand prenatal maternal antibiotic therapy was shown to be protective against systemic MSSA infections (Table 3).

## Discussion

The detailed evaluation of the here outlined NICU outbreak can teach three major lessons: firstly, a NICU outbreak can successfully be managed. Secondly, adequate nurse stuffing is crucial to sufficiently control an outbreak. Thirdly, non-resistant bacteria are likely to cause serious invasive infections in neonates which may be underappreciated, especially in the light of the widespread fear of multidrug-resistant organisms. Specifically, we characterize this NICU outbreak involving multi-resistant and non-resistant bacteria such as VRE and MSSA regarding molecular features, epidemiology, potential risk factors, effectivity of outbreak control measures and specific bacterial pathogenicity. This study provides the first longitudinal data on the effect of nurse staffing on nosocomial infections in a single tertiary care NICU center emphasizing the importance of adequate staff-patient balance.

Unfortunately, substantial nurse understaffing in NICUs is a common event. Cross-sectional studies have demonstrated nosocomial infection rates in understaffed NICUs to be significantly increased^17^. In consequence, most clinical guidelines recommend minimum staff-patient ratios of 1:4 for special care, 1:2 for high-dependency care and even 1:1 for intensive care patients^16^,^18^. In this report, we demonstrate that nurse understaffing was clearly associated with a substantial increase in neonatal nosocomial infections (Figs 1 and 2). Importantly, this was not restricted to very low birth infants but also included more mature neonates. Our observations were in line with data from several studies investigating the impact of organization and management factors on infection control in adult patient populations. These reports were able to link infection rates to workload, in terms of nurse staffing, bed occupancy and patient turnover^19^. Moreover, nurse staffing was shown to inversely correlate with the risk of nosocomial complications and the length of hospital stays in these patient populations. Hence, favorable staff-patient ratios will not only result in better clinical outcome but also – in a longer-term perspective – in medical cost saving, improved national productivity and ultimately, lives saved^20^. In fact, outbreaks of extended spectrum beta-lactamase-producing *Enterobacteriaceae* have recently been shown not only to be an alarming indicator of suboptimal patient care but also to be associated with significant mortality and prolonged disruption of the neonatal intensive care service^21^.

With respect to critical trigger factors involved in this outbreak, the data from this study strongly suggests that health care worker assisted transmission e.g. specifically understaffing related poor hand hygiene and clinical procedures significantly contributed to the outbreak. Even though stuff hand hygiene measures were not specifically recorded for this study, the VRE cluster analysis demonstrating tight clonal relationship (Fig. 3) supports this hypothesis. Moreover, this mode of transmission is not only the most likely one but also in line with similar previous analyses^22^,^23^. In fact, a meta-analysis revealed transmission from personnel to patients to be likely in as much as 63 (93%) of 68 studies that undertook genotyping^24^. As expected, we observed a reduction in colonization rates upon the introduction of hand hygiene audits and hand hygiene teaching sessions during this period. However, such measures alone or even in combination with cohorting of colonized infants, barrier nursing, staff movement restrictions, screening of healthcare workers and extensive environmental sampling were not sufficient to control the situation. Only after recruitment of adequately trained nursing staff were we able to control the outbreak and subsequently, both colonization and infection rates dropped to a pre-outbreak level. This is in line with the findings of a large randomized multicenter trial demonstrating that culture-based active surveillance for MRSA and VRE and the expanded use of barrier precautions alone was not sufficient to reduce transmission of MRSA and VRE in adult intensive care units^25^. Thus, this strongly underscores the essential importance of adequate staff-patient ratios as constituted by the national guidelines^16^,^18^. Noticeably, when compared to most reports on microbial outbreaks on NICUs that usually describe one specifically causative pathogen^26^,^27^,^28^ the here described outbreak involved multiple different organisms. Furthermore, this outbreak was unusually long-lasting covering a period of almost 9 months. Most probably, both these effects are associated with the insufficient staff-patient ratio in our unit which was ultimately due to the change in hospital management. To our knowledge no prior study reported a similarly long-lasting period of nurse understaffing in a tertiary care NICU.

Importantly, we made an intriguing discovery with respect to clinical relevance of specific pathogens for the affected patients. Whereas, PFGE typing of the VRE isolates revealed important virulence factors related to adhesion and capacity to cause infection in hosts (*esp, hyl*) and also to acquired resistance determinants (*vanA*) to both glycopeptides, there was not a single infection due to VRE during the entire outbreak. In contrast, a substantial number of patients colonized with MSSA became symptomatically infected. Apart from minor limited infections such as conjunctivitis this also included serious invasive disease such as pulmonary abscesses and osteomyelitis or bacteremia. Interestingly, whereas screening for MRSA and VRE is part of most national guidelines^29^,^30^, MSSA does neither entail equal prevention nor treatment measures. This is reflected by the fact that there are only very few recent reports on MSSA infection in neonates^31^,^32^, even though mortality and morbidity did not differ between MRSA and MSSA infection in VLBW infants^33^. Most probably, in the alarming light of multidrug-resistant strains such as VRE and MRSA, MSSA may remain unjustly under-recognized as a serious pathogen for neonates. Moreover, screening and prevention strategies only focusing on multi-resistant strains and MRSA in particular are potentially short-sighted and likely to spotlight only a part of the problem instead of illuminating the big picture of microbial challenges on modern NICUs.

In summary, with this first longitudinal data on a bacterial outbreak in a single tertiary care NICU center we provide clear evidence for the effect of nurse staffing on nosocomial infections emphasizing the importance of adequate staff-patient balancing. Moreover, our data strongly suggest that infection and colonization with non-resistant staphylococcus aureus in neonates is a serious problem – possibly even more than VRE and MRSA as the significance of MSSA in neonates currently might be under-recognized due to the ever-present discussion on MDROs.

## Additional Information

**How to cite this article:** Hensel, K. O. *et al*. Nursing staff fluctuation and pathogenic burden in the NICU - effective outbreak management and the underestimated relevance of non-resistant strains. *Sci. Rep.* **7**, 45014; doi: 10.1038/srep45014 (2017).

**Publisher's note:** Springer Nature remains neutral with regard to jurisdictional claims in published maps and institutional affiliations.

## Figures and Tables

**Table 1:** Epidemiological characteristic of MSSA, MDRO and VRE colonized patients.

	VRE total (n = 10)	MDRO (n = 9)	MSSA (n = 71)	p-value
Gestational age (weeks)	30.4 (28.2–32.7)	26.0 (23.8–28.2)	32.6 (31.5–33.7)	0.12
Birth weight (gramm)	1500 (1094–1900)	785 (559–1012)	2040 (1820–2261)	**0.04**
Age of the mother (years)	30.8 (27.2–34.4)	26.8 (22.3–31.3)	30.3 (29.0–31.7)	**0.04**
SGA	10%	33%	12%	0.26
Male gender	50%	100%	60%	0.36
Singeltons (%)	60%	67%	72%	0.58
progression to infection	0%	44%	32%	**0.02**

**Table 2:** Risk factors for progression from MSSA colonization to infection (p-values refer to the comparison of MSSA infection versus colonization).

	MSSA total (n = 71)	MSSA infection (n = 23)	MSSA colonization (n = 48)	p-value
Gestational age (weeks)	32.6 (31.5–33.7)	30.4 (28.6–32.2)	34.0 (32.8–35.3)	**0.002**
Birth weight (gramm)	2040 (1820–2261)	1632 (1277–1986)	2260 (1961–2560)	**0.02**
Age of the mother (years)	30.3 (29.0–31.7)	31.2 (28.9–33.4)	29.9 (28.2–31.7)	0.56
SGA	12%	12%	12%	0.64
Male gender	60%	36%	70%	**0.004**
Singeltons (%)	72%	64%	75%	0.29
prenatal steroids (<1500 g)	79%	75%	82%	0.69
prenatal antibiotics (<1500 g)	48%	25%	71%	**0.01**

**Table 3:** Epidemiological risk factors for systemic MSSA infection.

	Local infection (n = 17)	Systemic infection (n = 8)	p-value
Gestational age (weeks)	32.0 (30.6–34.9)	28.0 (24.0–32.0)	**0.02**
Birth weight (gramm)	1932 (1507–2358)	1183 (560–1806)	**0.01**
Age of the mother (yrs)	31.2 (28.2–34.2)	31.4 (27.2–35.1)	1.0
SGA	6%	33%	0.11
Male gender	33%	56%	0.41
Singeltons (%)	44%	11%	0.19
prenatal antibiotics (<1500 g)	56%	13%	**0.04**
prenatal steroids (<1500 g)	25%	75%	**0.02**
